# Transition of mild cognitive impairment to Alzheimer’s disease: Medications as modifiable risk factors

**DOI:** 10.1371/journal.pone.0306270

**Published:** 2024-08-14

**Authors:** Ying Wang, Mingfei Li, Dominique Haughton, Lewis E. Kazis

**Affiliations:** 1 Department of Mathematical Sciences, Bentley University, Waltham, Massachusetts, United States of America; 2 School of Computing and Data Science, Wentworth Institute of Technology, Boston, Massachusetts, United States of America; 3 Geriatric Research Education and Clinical Center, Bedford VA Healthcare System, Bedford, Massachusetts, United States of America; 4 Center for Healthcare Organization and Implementation Research, Bedford VA Healthcare System, Bedford, Massachusetts, United States of America; 5 Affiliated Researcher, Université Paris 1 (SAMM), Paris, France; 6 Affiliated Researcher, Université Toulouse 1 (TSE-R), Toulouse, France; 7 Department of Health Law, Policy and Management, Boston University School of Public Health, Boston, Massachusetts, United States of America; 8 Rehabilitation Outcomes Center (ROC), Spaulding Rehabilitation Hospital and Harvard Medical School, Boston, Massachusetts, United States of America; Ehime University Graduate School of Medicine, JAPAN

## Abstract

**Background:**

Mild cognitive impairment (MCI) is a pre-clinical stage of Alzheimer’s disease (AD). Understanding the transition probabilities across the disease continuum of AD, ranging from MCI to AD to Mortality is crucial for the economic modeling of AD and effective planning of future interventions and healthcare resource allocation decisions. This study uses the Multi-state Markov model to quantify the transition probabilities along the disease progression and specifically investigates medications as modifiable risk factors of AD associated with accelerated or decelerated transition times from MCI to AD, MCI to mortality, and AD to mortality.

**Methods:**

Individuals with MCI were identified from the National Alzheimer’s Coordinating Center between September 2005 and May 2021. A three-state Markov model was postulated to model the disease progression among three states: MCI, AD, and mortality with adjustment for demographics, genetic characteristics, comorbidities and medications. Transition probabilities, the total length of stay in each state, and the hazard ratios of the use of medications for diabetes, hypertension, and hypercholesterolemia (the known modifiable risk factors of AD) were evaluated for these transitions.

**Results:**

3,324 individuals with MCI were identified. The probability of developing AD after one year since the initial diagnosis of MCI is 14.9%. After approximately 6 years from the initial diagnosis of MCI, the probability of transitioning to AD increases to nearly 41.7% before experiencing a subsequent decline. The expected total lengths of stay were 5.38 (95% CI: 0.002–6.03) years at MCI state and 7.61 (95%CI: 0.002–8.88) years at AD state. Patients with active use of lipid-lowering agents were associated with significantly lower hazards of transitioning from MCI to AD (HR: 0.83, 95%CI:0.71–0.96), MCI to mortality (HR: 0.51, 95%CI:0.34–0.77), and AD to mortality (HR: 0.81, 95%CI:0.66–0.99).

**Conclusions:**

Results suggest that lipid-lowering agents may confer a protective effect, delaying the onset of AD. Additionally, lipid-lowering agents indicate a favorable association with a longer survival time.

## Introduction

Mild cognitive impairment (MCI) is considered to be a transitional state between normal aging and early Alzheimer’s disease (AD) [1]. MCI is a phase that provides an opportunity for early detection and intervention before significant neurodegeneration.

MCI is defined as an impaired cognitive state that does not meet the criteria for the diagnosis of dementia [2, 3]. Although the likelihood of transition from MCI to any form of dementia has been suggested to be 3 to 5 times higher than those transitions from unimpaired cognition, not all MCIs result from pathologic changes from AD [4, 5]. Other conditions, such as cardiovascular diseases, hypertension, diabetes, depression, anxiety, hypercholesterolemia, and smoking [6–10] have been found to contribute to cognitive dysfunction and to associate with MCI. While few subjects with MCI recover to near-unimpaired conditions, many remain stable with MCI [11]. In clinical practice, to determine whether someone with MCI may convert to AD, a clinician rules out other causes that account for cognitive changes, such as depression and related medications that may be associated with conversion from MCI to AD [12]. As such, it is important to understand how MCI transitions to AD.

Previous research reports a wide difference in the transition probabilities from MCI to AD, ranging from 1% to 34% per year [13–17]. This wide range reflects a mix of studies with small sample sizes, variations in the definition of MCI, limitations in the study design and analytic methodologies used [15, 16]. Other factors reported to affect the estimated conversion rate included the recruitment source, follow-up years, accounting for loss to follow-up, demographic/genetic/biomarker characteristics, and the lifestyles of the patients [17–19].

Epidemiological inquiries have revealed numerous modifiable comorbidity risk factors associated with the transition from MCI to dementia/AD. Effectively managing these modifiable comorbidity risk factors hold considerable promise for reducing transitions to AD. Hence, it is necessary to ascertain whether the utilization of FDA-approved medications targeting these recognized comorbidity risk factors of AD might play a role in its disease trajectory.

This study examines the transitions from MCI to AD and ultimately to mortality with a Multi-state Markov model. Our research uniquely explores the impact of medication usage for AD modifiable risk factors, specifically antihypertensive agents, lipid-lowering agents, medications for diabetes, and controls for medications addressing AD symptoms. These variables have not been previously included in Multistate Markov studies.

## Methods

### Study design

This study uses data from the National Alzheimer’s Coordinating Center (NACC) between September 2005 and May 2021. The investigated cohort includes individuals with clinically diagnosed MCI. Standardized criteria are utilized for the clinical diagnosis of individuals as cognitively unimpaired or with MCI, AD, or other dementias [20]. The analysis includes three stage states throughout the AD progression, that is, MCI, AD, and mortality. MCI is the initial state: AD is the first endpoint and mortality is the second endpoint/the absorbing state.

The objectives of this study are: (1)modeling the transition probabilities between states (MCI, AD, and Death), and examining the duration in each of the three states. (2) Evaluating the hazard risk of medications (antihypertensive agents, lipid-lowering agents, medication for diabetes) for managing known comorbidity risk factors of AD for each of the transitions.

### Data source and study population

We used data from the NACC, which adopted a retrospective, standardized, and longitudinal clinical evaluation of the subjects in the National Institute on Aging’s ADRC Program [21]. Data are collected by trained clinicians and clinic personnel from participants and their co-participants (e.g. family members or close friends). All AD/MCI diagnoses are made by either a consensus team or a physician who conducted the examination.

The population of this study is those who had been diagnosed with MCI and had at least two visits during a period between September 2005 and May 2021. Patients with extensive missing information on comorbidities were not included. Patients with MCI diagnosis after AD diagnosis were excluded from our study population. With the inclusion and exclusion criteria mentioned above, we obtained 3,324 qualified MCI patients.

Bentley University’s IRB (2023030) approved this study in accordance with the requirements set forth in 45 CFR 46.104(d)(4) for secondary research for which consent is not required.

### Study variables

#### Outcome variables

The outcome variables are the times to transition from state to state. For example, the transition time from state MCI to state AD is defined as the time from the first MCI diagnosis to the first AD diagnosis.

#### Medications that may impact transition times

The investigated medications included antihypertensive agents, lipid-lowering agents, and medications for diabetes. We also included the medication for AD symptoms as a control variable. The use of medication(s) was defined as “current use” if the patient reported the use of a medication within two weeks before the visit. The possible associations of the baseline use of medications under study with all transitions were evaluated.

#### Covariates

Demographic characteristics, APOE genotype, several known comorbidity risk factors at baseline, and the baseline use of medication for addressing AD symptoms were adjusted in the model. Demographic characteristics included age, gender, race, and ethnicity. The comorbidities included cardiovascular diseases (heart attack, congestive heart failure, stroke, Transient ischemic attack), diabetes, hypertension, hypercholesterolemia, seizures, vitamin B12 deficiency, thyroid, and the substance use of alcohol. All comorbidities were self-reported comorbidities collected by trained clinicians. All comorbidities include values of either “recently present” or “not present”. A disease was “recently present” if it was active in the past year or it required active management, and was consistent with information obtained from the subject and co-participant interview.

### Statistical analysis

Descriptive statistics included demographic characteristics, APOE genotype, and baseline comorbidities for the study cohort.

**Fig 1:**
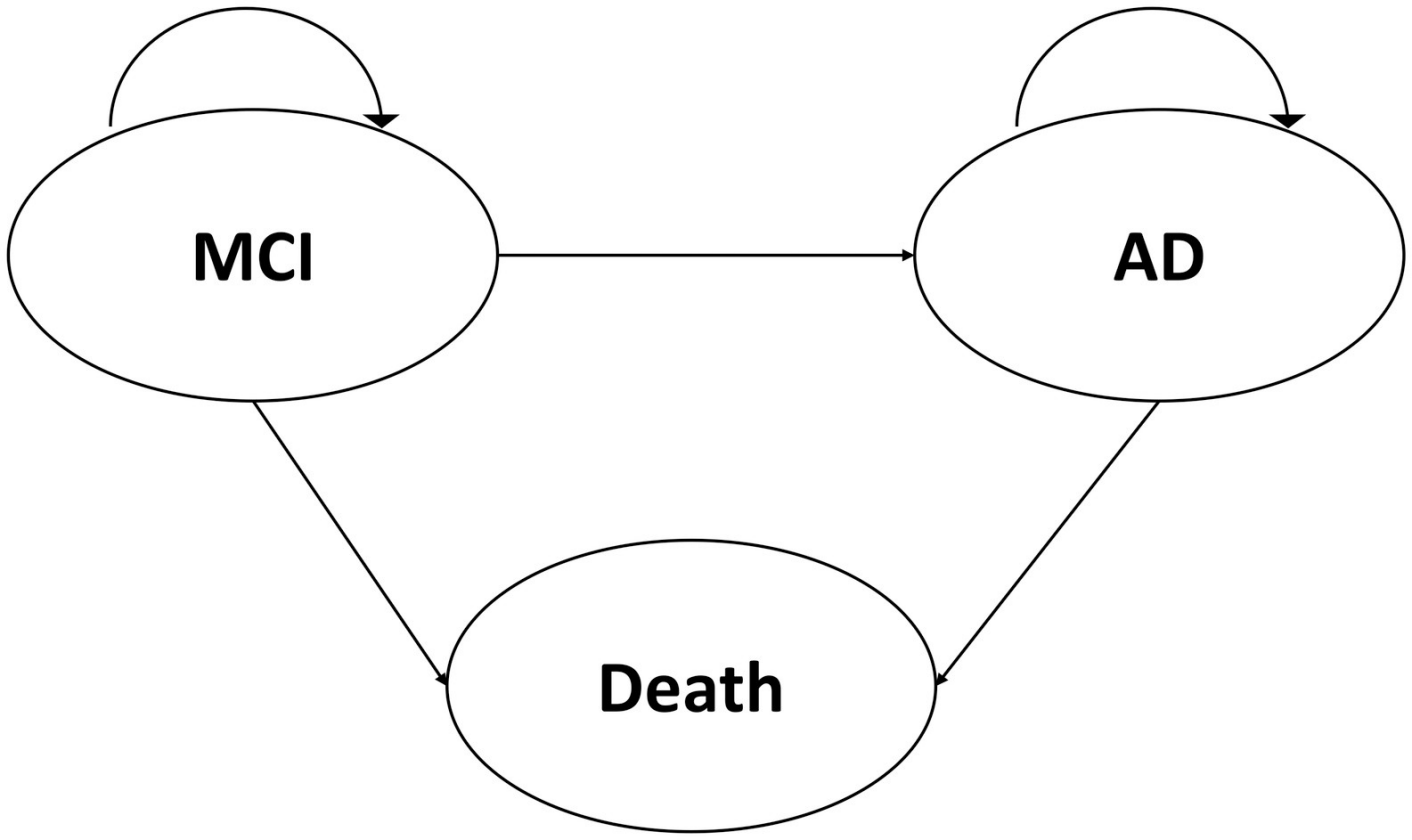
Three-state Multistate Markov model. The graph illustrates the three-state structure of the Multistate Markov model.

The Multi-state Markov model is an advanced approach that describes a stochastic process where more than one destination state exists; an estimation of the transition rates among multiple states simultaneously [22] are given. An important advantage of this method is that it can be implemented to accommodate intermittent observations, which are very common for longitudinal studies [23]. Covariates are fitted in the form of the Cox regression model [22, 24]. We postulate a three-state Markov Cox Regression model to capture the disease progression from MCI to AD to death (Fig 1). To be specific, we used Multistate Markov models to 1) Estimate the transition probabilities for all transitions: MCI-MCI, MCI-AD, MCI-death, AD-AD, and AD-mortality, as well as the total length of stay in each state; 2) Evaluate the hazard risk of the use of medications for the known comorbidity risk factors for all transitions. The model was controlled for possible confounding effects with demographics, genetic characteristics, comorbidities, and use of medication for AD symptoms. Transition intensity, transition probabilities, the total length of stay at each state, and the hazard ratios (HR) of investigated medication usage for each transition with 95% confidence intervals (CI) were reported. The R-package ’msm’ was used to conduct the multistate Markov model analysis [25].

## Results

A total of 3,324 MCI patients were included in the analysis. The average age at the initial visit was 73.1 ± 9.6 years. 50.2% of the study subjects were male, and 49.8% were female. The majority were white (80.6%) and non-Hispanic (91.2%). The most common APOE genotype was e3, e3 (accounting for 45.3% of the study subjects), followed by e3, e4 (23.4%), e3, e2 (9.1%), e4, e4 (4.2%), e4, e2 (2.3%), and e2, e2 (0.5%) (Table 1).

**Table 1:** Study subjects’ demographics and APOE genotype profile.

		Mean (SD)
Age at the initial visit		73.1 (9.6)
		Count (%)
**Gender**	Male	1667 (50.2%)
	Female	1657 (49.8%)
**Race**	White	2680 (80.6%)
	Black or African American	487 (14.7%)
	Asian	69 (2.1%)
	American Indian or Alaska	23 (0.7%)
	Other/Unknown	65 (2%)
**Ethnicity**	Not Hispanic or Latino	3031 (91.2%)
	Hispanic or Latino	282 (8.5%)
	Unknown	11 (0.3%)
**APOE**	e3, e3	1505 (45.3%)
	e3, e4	779 (23.4%)
	e3, e2	302 (9.1%)
	e4, e4	141 (4.2%)
	e4, e2	78 (2.3%)
	e2, e2	16 (0.5%)
	Unknown	503 (15.1%)

Hypertension (53.3%) and hypercholesterolemia (52.6%) were the highest prevalence for the study cohort, followed by diabetes (15.6%) and thyroid disease (15.3%) (Table 2). The prevalence for cardiovascular diseases, vitamin B12 deficiency, seizures, and substance abuse of alcohol were 7.0%, 3.3%, 1.3%, and 0.8% respectively (Table 2).

**Table 2:** Baseline comorbidity burden of the study subjects.

	Yes	No
**Hypertension**	1772 (53.3%)	1552 (46.7%)
**Hypercholesterolemia**	1749 (52.6%)	1575 (47.4%)
**Diabetes**	517 (15.6%)	2807 (84.4%)
**Thyroid**	507 (15.3%)	2817 (84.7%)
**Cardiovascular Diseases**	234 (7%)	3090 (93%)
**Vitamin B12 Deficiency**	110 (3.3%)	3214 (96.7%)
**Seizures**	44 (1.3%)	3280 (98.7%)
**Alcohol Abuse**	28 (0.8%)	3296 (99.2%)

59.5% of the study subjects were using antihypertensive medications of any type at the baseline (Table 3). The use of lipid-lowering medications, FDA-approved medications for AD symptoms, and diabetes medications at baseline were 46.1%, 16.8%, and 12.6%, respectively (Table 3).

**Table 3:** Baseline medication usage of the study subjects.

	Yes	No
**Antihypertensive agent**	1979 (59.5%)	1345 (40.5%)
**Lipid-lowering medication**	1532 (46.1%)	1792 (53.9%)
**Medication for Alzheimer’s disease symptoms**	560 (16.8%)	2764 (83.2%)
**Diabetes medication**	419 (12.6%)	2905 (87.4%)

### Transition intensity, transition probability, and total length of stay at each state

3,324 patients diagnosed with MCI were included in the study. Of these, 1,694 progressed to AD, while 1,630 did not progress to AD. Among those who progressed to AD, 1,147 subsequently died. Of the patients who did not progress to AD, 1,083 died.

**Fig 2:**
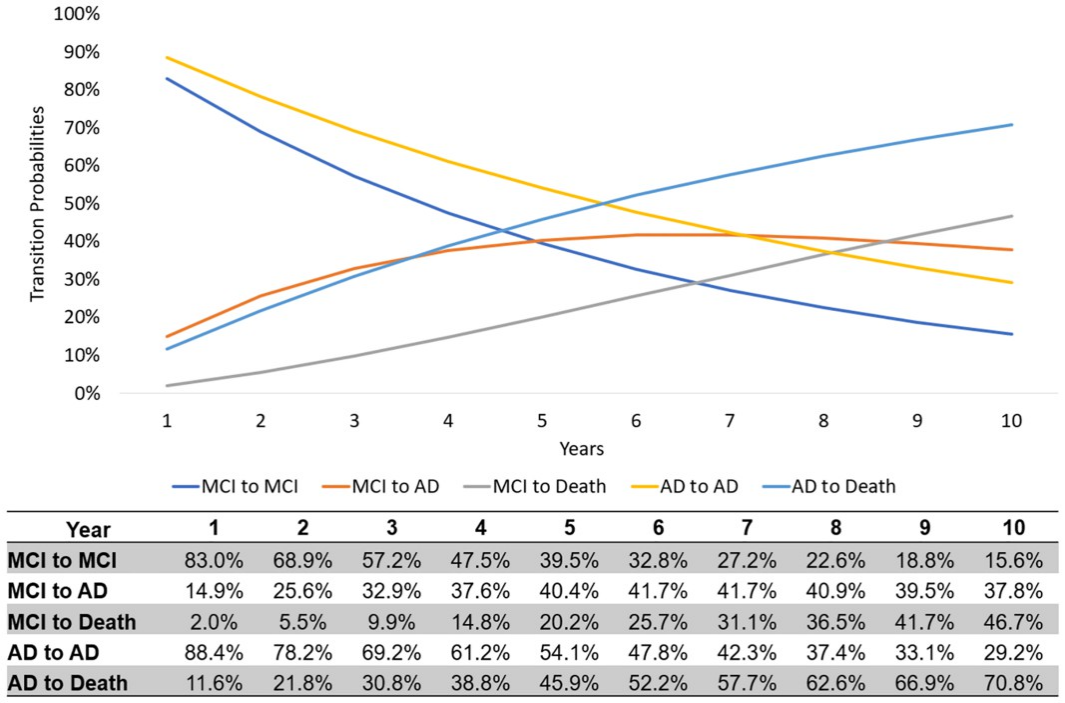
Estimated transition probabilities across 10 years. The graphical representation showcases the transition probabilities among states, derived from the estimated transition intensity, over a 10-year timeframe. The timeframes of years represent the time since the preceding state.

The hazard rate for MCI to AD was 0.174 (95% CI: 0.164, 0.185) while the hazard rate from AD to Death was (0.123, 95% CI: 0.110, 0.138) (Table 4). The probability of transition from MCI to AD after one year past the initial diagnosis of MCI is 14.9% (Fig 2). This probability increases to 41.7% after approximately six years from the initial MCI diagnosis, followed by a subsequent decrease. The transition probability to AD for five years and ten years after the initial diagnosis of MCI is 40.4%, and 37.8%, respectively (Fig 2). The probability of remaining as MCI patient after one year, five years, and ten years of the initial MCI diagnosis is 83.0%, 39.5%, and 15.6%, respectively (Fig 2). The probability of transitioning from the MCI state to death after one year, five years, and ten years since the initial diagnosis of MCI is 2.0%, 20.2% and 46.7%, respectively (Fig 2). The probability of transitioning from the AD state to death after one year, five years, and ten years of the initial AD diagnosis is 11.6%, 45.9%, and 70.8%, respectively (Fig 2).

**Table 4:** Transition intensities of Multistate Markov model.

	MCI	AD	Death
**MCI**	-0.186* (-0.366, -0.095)	0.174 (0.164, 0.185)	0.012 (0.000, 436.056)
**AD**	NA	-0.123* (-0.138,-0.110)	0.123 (0.110, 0.138)

Notes: The negative values indicate the outflow of the patients from MCI or AD. The value of outflow from MCI (-0.186) equals the value of inflow of from MCI to AD and MCI to Death (0.174+0.012). The value of outflow (-0.123) from AD to Death equals the value of inflow from AD to Death (0.123).

A patient is estimated to spend 5.38 (95% CI: 0.002–6.03) years with MCI, and 7.61 (95% CI: 0.002–8.88) years with AD after adjusting for demographics, APOE genotype, comorbidities, and medication use.

### Effect of medication use on the risk of progression from MCI to AD

The Multistate Markov model identified that patients with active use of any lipid-lowering agents were significantly associated with a delayed progression to AD, after controlling for demographic, genetic characteristics, the comorbidity profile of the patient, and the use of medication for AD symptoms (Table 5).

**Table 5:** Association of use of medications for known comorbidity risk factors of AD with the risk (hazard ratios) of transitioning.

Medication	MCI to AD	MCI to Death	AD to Death
**Anti-hypertensive agents**	0.88 (0.75, 1.02)*	1.29 (0.86, 1.94)	0.93 (0.75, 1.15)
**Lipid-lowering agents**	**0.83 (0.71, 0.96)**	**0.51 (0.34, 0.77)**	**0.81 (0.66, 0.99)**
**Medication for Diabetes**	1.01 (0.74, 1.37)	1.78 (0.76, 4.13)	1.21 (0.79, 1.87)

Notes: Hazard ratios with significant p-values are highlighted in bold.

* Anti-hypertensive agents show a trend towards significance although not significant at p = 0.05 level with a confidence interval of 0.75 to 1.02.

Patients with active use of an anti-hypertensive medication displayed a non-significant trend of 12% reduction in the hazard rate (HR: 0.88, 95%CI:0.75–1.02) of transitioning from MCI to AD, compared with those without active use of anti-hypertensive medications (Table 5).

Patients with active use of lipid-lowering agents were associated with significantly lower hazards of transitioning from MCI to AD (HR: 0.83, 95%CI:0.71–0.96), MCI to Death (HR: 0.51, 95%CI:0.34–0.77), and AD to Death (HR: 0.81, 95%CI:0.66–0.99) (Table 5).

## Discussion

The dynamic nature of AD progression makes it challenging to precisely predict its time course. In this study, we analyzed data obtained from the NACC spanning from September 2005 to May 2021 and used the Multistate Markov model to examine the transition probabilities between stages throughout the AD progression (MCI, AD, and Mortality) and the time course for a patient at each stage with large-scale longitudinal data. Our estimations were adjusted for demographics, APOE genotype, comorbidities, and medication use. In addition, our research focuses on the impact of medication utilization targeting modifiable factors of AD associated with the progression of the disease. The medications under evaluation were those approved by the FDA and comprised antihypertensive agents, lipid-lowering agents, and diabetes medications.

The Multi-state Markov model [26] was used in estimating the transition probabilities among multiple states simultaneously and investigated the association of medication use and their transitions. In recent years, this approach has been introduced to study the progression of AD [27–31].

The Multistate Markov model identified that the instantaneous rate of transition from MCI to AD (hazard rate from MCI to AD: 0.174, 95% CI: 0.164, 0.185) is higher than the instantaneous rate of transition from AD to Death (hazard of movement from AD to Death: 0.123, 95% CI: 0.110, 0.138). The instantaneous rate of transition from AD to Death is 10.3 times higher (0.123/0.012) than the instantaneous rate of transition from MCI to Death. The estimated time for a patient with MCI was 5.38 (95% CI:0.002–6.03) years, and with AD was 7.61 (95% CI: 0.002–8.88) years. The average life expectancy of AD patients estimated from our study was consistent with previous studies, which suggested that the life expectancy of individuals with AD may range from 3 to 10 years, and those who receive a diagnosis in their 60s and early 70s may anticipate a median lifespan ranging from 7 to 10 years [32].

The variability in life expectancy among individuals with AD can be attributed to a range of contributing factors, including gender, the age at which AD manifests itself, and the lifestyle of the individuals [33–35]. A gender-based disparity was revealed in a 15-year follow-up study, with male AD patients experiencing a shorter mean survival period of 8.20 years (95% CI 7.82–8.57), while their female counterparts exhibited a notably longer mean survival duration of 9.22 years (95% CI 8.90–9.53) [34]. We arrived at a comparable estimation for this study. Another investigation elucidated that the median survival period was subject to substantial variation depending on the age at which patients received their AD diagnosis [33]. Specifically, individuals diagnosed at the age of 65 years displayed a median survival time of 8.3 years, whereas those diagnosed at the age of 90 years confronted a significantly reduced median survival time of 3.4 years [33]. A healthy lifestyle has been postulated to be associated with a longer total life expectancy and fewer years lived with Alzheimer’s dementia across the lifespan [35]. This study shows shorter estimated life expectancy for individuals with AD, ranging from 1.4 to 6.8 years depending on their gender, age, and the level of adherence to a healthy lifestyle. Comparisons across studies are difficult given different study designs and analyses. Although pertinent, one should exercise caution when making direct comparisons among results. These findings underscore the intricate interplay of various factors in molding the lifespan of individuals with AD, and the necessity to employ diverse approaches for validating estimations across different cohorts.

The active use of lipid-lowering agents for individuals with MCI had significantly lower hazards of transitioning from MCI to AD and from MCI to mortality. These results suggest an overall benefit of cholesterol management with lipid-lowering agents for individuals with MCI, i.e., association with prolonged survival time and lower risk of the occurrence of AD. The association between cholesterol levels and AD remains unclear as to whether this is related to the association with the brain or circulating cholesterol metabolism, or both [36]. It is anticipated that acute treatment with statins is unlikely to affect brain homeostasis over a short period of time or improve cognitive functioning. Several studies revealed a positive association between low levels of circulating low-density lipoprotein cholesterol (LDL-C) and cognitive decline, as well as the risk of AD [37–40]. Recent findings found LDL-C in the pathophysiology processes of AD independent of APOE [41, 42]. Our results suggest an overall benefit of cholesterol management with lipid-lowering agents for those with MCI.

In our study, we did not detect a significant association between active use of anti-hypertensive agents among individuals with MCI and a reduced transition from MCI to AD at the 5% significance level. It is noteworthy that the upper bound of the 95% confidence interval (1.02) is in close to 1. Future research with data sets that provide larger sample sizes are needed. The definitive mechanisms through which anti-hypertensive agents afford protection against AD is not known and whether the effects mainly work through blood pressure-dependent effects or blood pressure-independent effects, or both. Prospective studies on blood pressure-independent effects revealed mixed results [43–47]. Several studies and meta-analyses suggested that angiotensin II receptor blockers (ARB) may have superior effects on cognitive function than other antihypertensive agents [43, 48–52]. A clear understanding of the underlying mechanism is necessary and important for future work [45].

This study has several limitations. First, we did not have sufficient brain imaging or biomarker studies that would contribute to the accuracy of the clinical diagnosis of MCI or AD. Second, we investigated the general use of medications among the study cohort, the heterogeneity of the medications within each category was not specific. Future studies can differentiate anti-hypertensive/lipid-lowering agents by classes or chemical properties, etc. Third, the Multistate Markov model for this analysis is a time-homogeneous model, which assumes a constant instantaneous transition rate. Disease progression may not be constant throughout the course of the disease. Despite these limitations, our study provides some contributing evidence regarding an understanding of patients who transition from MCI to AD, MCI to Death, and AD to Death in a clinical-based cohort. Future studies can be carried out in other population-based studies in the future.
